# Randomised controlled pilot trial to assess effect of electrical stimulation of weak pelvic floor muscles

**DOI:** 10.1007/s00404-024-07389-2

**Published:** 2024-03-29

**Authors:** Ingeborg Hoff Brækken, Tove K. L. S. Villumstad, Natalie Michelle Evensen

**Affiliations:** 1 Department of Research and Innovation, Akershus University Hospital, The Pelvic Floor Centre, Lørenskog, Norway; 2 Health Department Northern Follo Municipality, Kolbotn Physiotherapy Institute, Kolbotn, Norway; 3 Division of Medicine, Physiotherapy, Akershus University Hospital, The Pelvic Floor Centre, Lørenskog, Norway; 4 Health Department Northern Follo Municipality, Centrum Physiotherapy Ski DA, Ski, Norway

**Keywords:** Pelvic floor muscle training (PFMT), Electrical stimulation, Pelvic floor muscle (PFM) strength

## Abstract

**Introduction and hypothesis:**

Pelvic floor muscle training (PFMT) has level 1A scientific evidence for the treatment of urinary incontinence and pelvic organ prolapse. Past studies, however, have often excluded women with very weak pelvic floor muscles (PFM). The aim was to investigate the hypothesis that intravaginal electrical stimulation (iES) improves PFM strength more than PFMT in women with weak PFM, and to use these results to calculate sample size required for a future large randomised controlled trial (RCT).

**Methods:**

This assessor-blinded pilot RCT had a two arm, parallel design with computer-generated Randomisation. Both groups were offered 12 one-to-one physiotherapy sessions over a 6-month period. The iES group received individual tailored electrical pulse parameters. The PFMT group received PFM exercises, with the addition of facilitation techniques at therapy sessions. A power calculator was used to calculate sample size.

**Results:**

Fifteen women were recruited. Eight were randomised to iES and 7 to PFMT. Two subjects dropped out of the iES group. Median age was 49 years (range 36–77) and parity 2.1 (range 1–3). Both groups showed increases in PFM strength measured by manometery (iES 12.3, SD 12.0 vs PFMT 10.0, SD 8.1) cmH_2_O. There was no significant difference between groups. With a power of 0.80 we need a sample size of 95 women in each group to detect a difference between groups.

**Conclusion:**

There was no significant difference between the groups in improvements in PFM strength. To detect a difference, we would have required 95 women in each group.

## What does this study add to the clinical work

**Table:** 

Both iES and PFMT improves PFM strength in women with weak PFM. A large-scale RCT is required to determine if one therapy intervention is more effective.	

## Introduction

Pelvic floor muscle training (PFMT) has level 1A scientific evidence for the treatment of urinary incontinence and pelvic organ prolapse (POP) [1–3]. Unfortunately, women who have very weak PFM have often been excluded from these studies. Approximately 30% of women are unable to contract their pelvic floor muscles (PFM), even after thorough individual instruction [4, 5]. Following instrumental vaginal deliveries, PFM strength has been found to be reduced by 54–66%, and endurance by 53% and 65%, respectively [6]. Both electrical stimulation (ES) and vaginal facilitation techniques can be used to facilitate PFM contraction [9]. Intravaginal electrical stimulation (iES) of weak PFM may be a better treatment approach than PFMT. There is currently little evidence to support this hypothesis. When commencing our pilot study, there had only been one randomised controlled trial (RCT) that had investigated the effect of iES on PFM strength in women with weak PFM [8]. Mateus-Vasconcelos et al. (2018) investigated 132 women with a PFM strength of 0 or 1 measured by vaginal palpation using the Modified Oxford Scale, where 33 women were randomised to iES [8]. They found that iES was no better than PFMT, but they likely did not use optimal electrotherapy parameters and only measured PFM strength with vaginal palpation. To our knowledge, any other studies that had investigated iES prior to 2020 included subjects that were not very weak [10, 11], had small study samples [9], no control group [9] or did not have subjects simultaneously voluntarily contract their PFM with the electrical current [8, 10, 11]. There is an urgent need for treatment options for women with weak PFM. The hypothesis of the present study was that iES is more effective than PFMT in improving PFM strength in women with very weak PFM.

## Methods

The primary aim of this study was to determine the change in PFM strength between women allocated to iES and PFMT groups, in order to calculate the sample size required to undertake a large RCT to establish if one treatment approach is superior to the other. Secondary aims were to:

1. Determine if there were changes in symptoms of pelvic floor dysfunction (PFD) between the groups.
2. Assess differences in patient perspectives and user participation between the groups.
3. Assess the characteristics of those women who achieve the greatest improvements in PFM strength with both interventions.

### Design

**Fig. 1:**
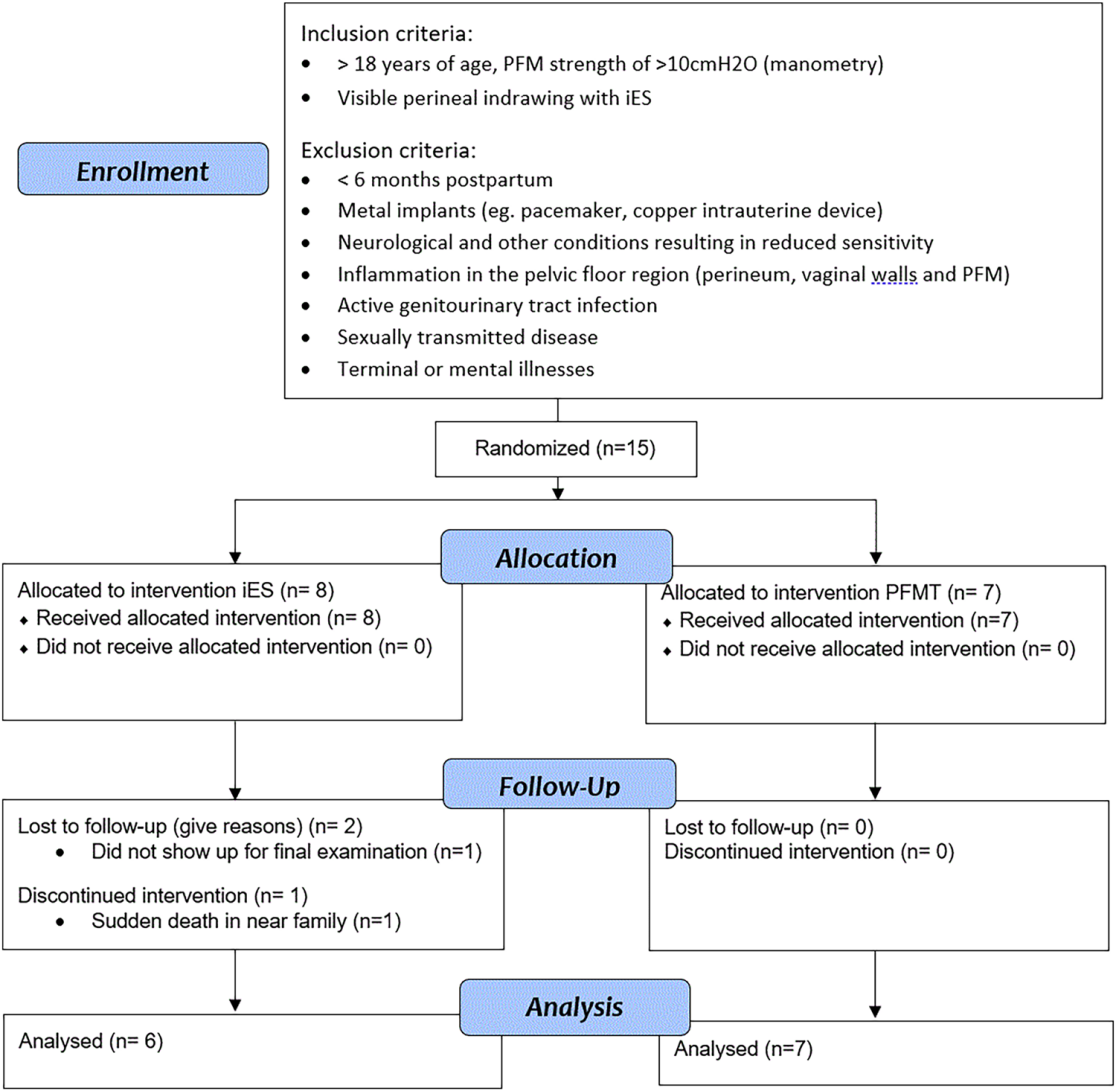
Participant flowchart

This study was an assessor-blinded, two-armed, pragmatic pilot RCT for participants receiving physiotherapy for PFD and who had weak PFM. Participants were recruited via medical clinics, gynaecologists, and midwives at health stations. Women who presented to physiotherapy with PFD and weak PFM strength were invited to participate in the study. Potential participants were provided with verbal and written information regarding the study and gave informed consent prior to baseline testing and allocation to treatment group. A computer-generated random number system with opaque sealed envelopes was used to randomise the women 1:1 to either iES or PFMT group (Fig. 1). The physiotherapists (PT) undertaking pre- and post-testing were blinded to group allocation. The treating PTs were not blinded to treatment allocation. All PTs involved in this study had at least 20 years of experience as a women’s health PT. This study was approved by The Norwegian Regional Committees for Medical and Health Research Ethics (Ref 125,707). If the present pilot study results in a full-scale RCT, it will be registered in the Clinical Trials Register.

### Participants

Women were eligible for inclusion in the study if they were over the age of 18 years, if their PFM strength was less < 10 cmH_2_0 as measured by manometry (Camtech, Oslo, Norway) and if they demonstrated visible perineal indrawing with iES. Exclusion criteria were: < 6 months postpartum; pregnant; metal implants (eg. pacemaker, copper intrauterine device); neurological and other conditions resulting in reduced sensitivity or pain in the pelvic floor region (perineum, vaginal walls and PFM); active genitourinary tract infection; sexually transmitted disease; inability to complete a questionnaire in Norwegian; and terminal or mental illnesses affecting the ability of the participant to follow instructions. This study aimed to recruit as many participants as possible from April 2021 until the end of 2022, with a minimum of six participants in each group (Fig. 1).

### Recruitment procedure

Prior to baseline testing, participants completed a 20-min questionnaire. The questionnaire contained questions related to demographics and socioeconomic variables, bladder function, vaginal symptoms, bowel function, and sexual symptoms [12–15]. Subjects underwent a full subjective and objective assessment with their treating PT and received instructions on PFM anatomy and how to correctly perform a PFM contraction. The ability to contract PFM was evaluated using visual observation and palpation [16, 17]. If after 1 week of practising recruiting PFM twice a day, subjects were still classified as being weak (grade 0 or 1 on the Modified Oxford Scale [17]), they were invited to participate in our study. Subjects who accepted the invitation were referred to another PT who tested PFM strength using a vaginal manometer (Camtech, Oslo, Norway). Weak PFM muscles were defined as a maximum voluntary contraction (MVC) below 10 cmH_2_0 and was a requirement to fulfil the inclusion criteria.

### Interventions

Both groups were offered 12 one- to one PT sessions (1 × 60 min, 11 × 30 min) and were instructed to perform daily home training, 7 days a week, for 6 months. A training log was used to record adherence to home training. We aimed to prescribe both groups the same training dosage but different training methods. All participants were advised to avoid straining with bowel motions and instructed on how to contract their PFM before and during increases in abdominal pressure (eg. coughing, sneezing, lifting), called ‘the knack’ [18].

#### iES group

**Fig. 2:**
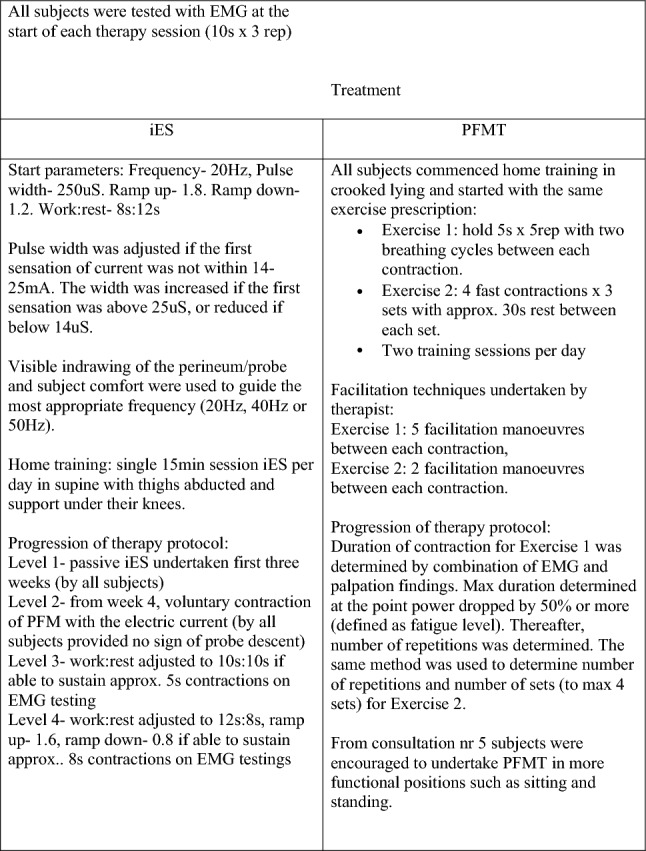
Testing and treatment protocol at treatment sessions

Subjects in the iES group used a NeuroTrac MyoPlus Pro muscle stimulation machine (Quintet, Bergen, Norway) once a day with either a 33 mm transverse diameter vaginal probe (Periform, Quintet, Bergen, Norway) or an anal probe of diameter 25mm (Anuform, Quintet, Bergen, Norway). The anal probe was chosen if POP made it difficult to keep a vaginal probe in place. Electrical stimulation parameters were tailored to each participant. Figure 2 shows the start parameters and progression of therapy.

#### PFMT group

The PFMT group undertook PFM exercises twice a day at home, with the addition of facilitation techniques at treatment sessions only. Progression was individualised according to a protocol with increasing contraction holding time, number of contractions and altering training positions (Fig. 2).

Muscle facilitation techniques were undertaken by the treating PT who applied a light stretch to levator ani, puborectalis, prior to each PFM contraction (Fig. 2). Vaginal palpation and EMG testing were used by the treating PT to guide progression with training.

### Outcome measures

#### Primary outcomes

**Fig. 3:**
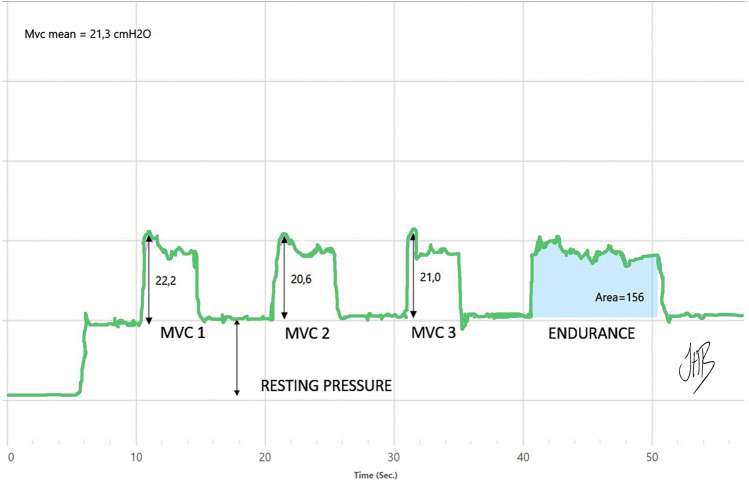
Vaginal manometry measurement for one participant showing vaginal resting pressure, pelvic floor muscle strength measured as three maximum voluntary contractions (MVCs) and pelvic floor muscle endurance

The primary outcome was PFM strength using a manometer (Mano, version 2.1, Camtech, Oslo, Norway). The manometer records pressure using a high-precision pressure transducer and a vaginal balloon, sized 67 × 17 mm. The balloon was placed 3.5 cm proximal to the vaginal introitus [19, 22]. PFM strength was defined by the average of three MVCs (cmH_2_O) (Fig. 3). PFM endurance was calculated as the work achieved during a 10 s work period (area under the curve, cmH_2_O, Fig. 3) [20, 21]. Vaginal resting pressure was defined as the vaginal pressure at rest with as little activation of the PFM as possible (cmH_2_O) (Fig. 3) [20]. The Camtech manometer is a reliable and valid measurement tool when used with simultaneous observation of inward movement of the catheter during PFM contraction [19, 20, 22]. It has demonstrated very good intra- and inter-reliability, with a minimal detectable change for interrater assessment of 8.7 cmH_2_O for vaginal resting pressure, 7.6 cmH_2_O for PFM strength, and 59.5cmH_2_O/s for muscular endurance [20].

#### Secondary outcomes

Changes in symptoms were evaluated by responses to standardised questionnaires for PFD, all of which have been tested for reliability, validity and responsiveness. The International Consultation on Incontinence Questionnaire (ICIQ)- Urinary Incontinence Short Form (ICIQ-UI SF) was used to detect changes in urinary incontinence [12], the ICIQ- vaginal symptoms (ICIQ -VS) evaluated symptoms of POP [13], ICIQ-bowel (ICIQ-B) assessed bowel function, and ICIQ- Flut Sex [15] evaluated sexual symptoms. Patient perspectives and adverse events were evaluated by a questionnaire and semi-structured interview all of which are described in the recruitment procedure. Any discomfort or side effects from intervention was asked for at each consultation and documented. Responders were defined as those who increased their PFM strength more than 10 cmH_2_O as measured by manometery, independent of their group allocation.

#### Adherence

Adherence to the intervention was recorded over the 6-month period using a training log. Participants were asked to document reasons for not undertaking training (for the purpose of determining potential side effects or technical issues).

### Statistics

Statistical analyses were performed using SPSS version 28. Data was first tested for normality using Kologorov–Smirnov. Results are given as frequencies and percentage for categorical data and means with standard deviation for continuous data. Differences between groups for categorical data were analysed with Person Chi-square test. For continuous variables, the Mann–Whitney U test was used for data that was not normally distributed. The level of significance was set to 0.05. The power calculator we used to calculate sample size used the following formula: *n* = (σ $\frac{z1-\mathrm{\alpha }/2+z1-\upbeta }{\upmu -\upmu 0}$)^2^, where σ is standard deviation, β is Type II error, µ true mean, µ_0_ is null hypothesis and Z = $\frac{\upmu -\upmu 0}{\upsigma /\sqrt{n}}$ [23, 24]. P values < 0.05 were considered significant.

## Results

Subject recruitment and data collection were undertaken between April 2021 and November 2022. A total of 15 women were assessed as eligible for participation in the study. Eight were randomised to iES and 7 to PFMT. There were two dropouts in the iES group. One withdrew due to a sudden death in the family and the other was lost to follow up just prior to post-testing. Statistical analyses were undertaken on the 13 women who completed post-testing (Fig. 1). Demographical data are presented in Table 1. Median age of the participants was 49 years (range 36–77) and parity 2.1 (range 1–3). At baseline mean PFM strength was 6.9 (SD 1.1) and 6.0 (SD 2.4) cmH_2_O in the iES and PFMT group respectively. There were no significant differences between groups at baseline (*p* = 0.392) (Table 1).

**Table 1:** Descriptive statistics and baseline values for women randomised to electrical stimulation (iES, *n* = 6) and pelvic floor muscle training (PFMT, *n* = 7). Results are given as frequencies and percentage for categorical data and means with standard deviation for continuous data

	iES	PFMT	*p* value
Higher education (> 4 years university)	3 (50.0%)	2 (28.6%)	0.429
Postmenopausal	4 (66.7%)	1 (14.3%)	0.053
Caesarean section	0	2 (28.6%)	0.161
Vacuum	1 (16.7%)	1 (14.3%)	0.906
Forceps	2 (33.3%)	0	0.097
Urinary incontinence	6 (100%)	7 (100%)	
Stress urinary incontinence	6 (100%)	6 (85.7%)	0.335
Urgency urinary incontinence	4 (66.7%)	6 (85.7%)	0.416
ICIQ-UI SF	10.2 (1.6)	9.7 (3.1)	0.754
Pelvic organ prolapse symptoms			
Vaginal bulging	1 (16.7%)	2 (28.6%)	0.612
Vaginal symptom score (ICIQ-VS)	15.7 (12.5)	11.1 (13.0)	0.538
Bowel dysfunction			
Bowel urgency *	4 (66.7%)	3 (42.9%)	0.391
Flatus incontinence *	2 (33.3%)	1 (14.3%)	0.416
Faecal incontinence *	3 (50.0%)	1 (14.3%)	0.164
Sexual dysfunction ICIQ-FlutSex	9.8 (13.5)	3.3 (4.1)	0.245
PFM function—manometry			
Maximal voluntary contraction (cmH_2_O)	6.9 (1.1)	6.0 (2.4)	0.392
PFM endurance (cmH_2_Osec)	26.3 (14.9)	41.9 (20.5)	0.152
Vaginal resting pressure (cmH_2_O)	27.1 (6.7)	32.9 (18.4)	0.480
PFM function—EMG			
Maximal voluntary contraction (µV)	14.0 (3.9)	14.4 (10.1)	0.921
Vaginal resting activity (µV)	7.4 (3.9)	5.9 (5.9)	0.693

^*^ Frequency of symptoms was graded: sometimes, most of the time or always

ICIQ-UI SF, The International Consultation on Incontinence Questionnaire—urinary incontinence short form; ICIQ-VS, The International Consultation on Incontinence Questionnaire–vaginal symptoms; ICIQ-FlutSex, The International Consultation on Incontinence Questionnaire–sexual symptoms; PFM, pelvic floor muscle; EMG, electromyography

### Change in PFM strength and endurance, and power calculations

Both groups showed increases in PFM strength from baseline that exceeded the minimal detectable changes found in the study by Tennfjord et al. [20] (iES 12.3, SD 12.0 vs PFMT 10.0, SD 8.1 cmH_2_O. No significant difference, however, was found between groups (2.3, 95%CI −10.0–14.7 cmH_2_O, *p* = 0.886). With a power of 0.80 (1 – β) and Type 1 error rate of 5% (α), we need a sample size of 95 women in each group to detect a difference between the two groups (Table 2). PFM endurance also improved in both groups (iES 95.7, SD 81.7 vs PFMT 73, SD 69.5 cmH_2_O). There was no significant difference between groups (22.7, 95%CI −69.5–114.9, *p* = 0.886) (Table 2).

**Table 2:** Changes in pelvic floor muscle (PFM) strength (maximal voluntary contraction, MVC), endurance and vaginal resting pressure, measured with Camtech manometer. Mean with 95% confidence interval

Change in:	iES group	PFMT group	Difference between groups	*p*-value	Sample size
MVC (cmH_2_O)	12.3 (−0.3–24.9)	10.0 (2.5–17.4)	2.3 (−10.0–14.7)	0.886	95
Endurance (cmH_2_Osec)	95,7 (9.9–181,4)	73.0 (8.7–137.3)	22.7 (−69.5–114.9)	0.886	74
Vaginal resting pressure (cmH_2_O)	−0.7 (−6.6–5.0)	−2.2 (−17.3–12.9)	1.5 (−13.9–16.8)	0.317	925

### Change in symptoms

Change in symptoms are presented in Table 3. Improvements in ICIQ-UI SF scores was found in both groups, with improvements tending to be a little better in the PFMT, but not reaching significance (3.7, 95% CI −0.4–7.9, *p* = 0.073). Participants in both groups reported fewer episodes of urinary incontinence (iES 67% and 57% PFMT). All subjects with POP symptoms reported improvement in quality of life in relation to vaginal symptoms with the difference between groups tending significance in favour of the PFMT group (2.5, 95% CI −0.3–5.3, *p* = 0.061). Sample size calculations for changes in urinary, vaginal bowel and sexual symptoms, based on ICIQ questionnaires, are presented in Table 3

**Table 3:** General improvements and specific improvements in bladder-, pelvic organ prolapse- (POP) and bowel- symptoms in the electrical stimulation (iES, *n* = 6) and pelvic floor muscle training (PFMT, *n* = 7) group

	iES	PFMT	Difference between groups with 95% CI	P-value	Sample size
General improvements					
Improved	6/6 (100%)	7/7 (100%)		NA	
Satisfied	6/6 (100%)	7/7 (100%)		NA	
Recommend others the same treatment	6/6 (100%)	7/7 (100%)		NA	
Improvement in bladder symptoms					
Change in ICIQ-UI SF score	−0.4 (SD−3.5)	−4.1 (SD− 3.2)	3.7 (−0.4–7.9)	0.073 ¤	12
Reduced frequency of UI (0–5)	4/6 (67%)	4/7 (57%)		0.529 *	
POP symptoms					
Change in ‘vaginal bulging’, for women initially having the symptom	0/1	1/2 (50%)		0.386 *	
Change in QoL vaginal score (0–10)	−1.5 (Range −5–1)	−4.0 (Range −7–−1)	2.5 (−0.3–5.3)	0.061 ¤	14
Bowel symptoms					
Change in bowel urgency (0–5)	0.2 (Range −1–1)	−0.6 (Range −2–2)	0.7 (−0.6–2.0)	0.117 ¤	
Change in QoL bowel score (0–10)	−1.5 (Range −5–0)	−2 (Range −6–0)	0.5 (−2.1–3.1)	0.767 ¤	335
Improvement in sexual function					
Change in ICIQ-Flut Sex score	−6.3 (SD−7.8) (*n* = 3)	−2.7 (SD 4.1) (*n* = 6)	3.7 (−12.7–5.4)	0.500 ¤	38

¤ Analysed with Mann–Whitney *U* test (category scales)

^*^ Tested with Pearson Chi-Square

CI, confidence interval; ICIQ-UI SF, The International Consultation on Incontinence Questionnaire—urinary incontinence short form; UI, urinary incontinence; QoL, quality of life; ICIQ-Flut Sex, The International Consultation on Incontinence Questionnaire–sexual symptoms

### User perspective and adverse events

All subjects in both groups (100%) reported improvements in their symptoms of PFD, that they were satisfied with the treatment, and that they would recommend the same therapy to others (Table 3). One subject reported pain of her vaginal lining after using iES for a period. She was advised to stop using the device for two weeks and then recommence using more lubricant. No pain or discomfort was reported thereafter. Two women had some technical difficulties with their NeuroTrac MyoPro Plus machine which they reported to their treating PT immediately.

### Responder analyses

There were three responders in each group. Responders (*n* = 6) increased their strength by 19.6 ± 6.9 cmH_2_O whereas non-responders (*n* = 7) increased by 3.5 ± 2.7 cmH_2_O (*p* = 0.001). Similarly, responders improved their PFM endurance more than non-responders (151.8 ± 41.2 vs 24.9 ± 23.9 cmH_2_O). At baseline non-responders were significantly weaker than responders (5.2 ± 1.7 vs 7.8 ± 1.0 cmH_2_O, *p* = 0.014) and had lower PFM endurance (24.3 ± 23.9 vs 19.8 ± 6.9 cmH_2_O/s, *p* = 0.035) compared to responders. We found no significant differences between or within the groups regarding age, adherence, or birth history. There was a trend towards significance of the non-responders having had larger babies than the responders (4149 g vs 3649 g, *p* = 0.222). A sample size of 16 participants in each group would have been required to a detect significant difference.

### Adherence

Adherence was very good for subjects in both groups, with no significant difference being found between groups. The number of days performing iES/PFMT was 154 of 187 (SD 31.8, 82%) and number of physiotherapy visits was 11.2 out of 12 (range 10–12, 93%).

## Discussion

Our study shows that both iES and PFMT can improve PFM strength, endurance and symptoms of PFD in women with weak PFM. Due to the small number of participants, however, we were unable to determine if iES was a more effective therapy than PFMT. Based on our results, a large-scale RCT with 95 patients in each group is required to answer this question.

Past studies investigating the effect of PFMT have often excluded women with weak or no voluntary contraction of PFM [1]. Women with weak PFM and damage to their pelvic floor support have a higher prevalence of UI and POP compared to women with good support. Such women may be at increased risk of developing PFD that not only significantly impacts on their quality of life but also on health care costs.

Pelvic floor muscle contraction involves complex neurophysiological processes. Vaginal palpation is a proprioceptive stimulus that can be used to facilitate correct voluntary contraction of the PFM [7]. Electrical stimulation applied intravaginally can also facilitate PFM contraction by means of stimulating the pudendal nerve and its branches and producing a reflex contraction. Both perineal rehabilitation methods may be beneficial in women who have difficulty contracting their PFM.

At the time of planning our study there was only one RCT by Mateus-Vasconcelos et al. (2018) that had investigated use of iES in patients with no ability to contract or very weak PFM [8]. They compared iES undertaken once a week for 8 weeks, with PFMT with facilitation techniques, with and without pelvic tilt. Intravaginal ES was found to be the least effective intervention. Subjects in the other two groups demonstrated a better ability to voluntarily contract their PFM following the intervention period and reported greater improvements on self-reported urinary incontinence. It is possible that iES was less effective as subjects in this group were specifically instructed not to voluntarily contract their PFM.

This is supported by a past consensus statement concluding that electrically stimulated muscle contractions alone are less effective at improving muscle strength compared with voluntary contractions [25].

It is currently not known what the optimal ES parameters are for women who have difficulty with voluntarily contracting their PFM [26]. Use of standardised protocols for specific conditions like SUI are not always optimal. The present study designed a protocol based on past studies that have undertaken iES [8, 27, 28] and clinical experience. Electrical stimulation parameters were individually tailored with the aim of achieving a good visible indrawing of the perineum that was considered comfortable for the participant, and a standardised protocol implemented for progression of therapy. To our knowledge, our study is the first to have undertaken iES of PFM on a daily basis over many months. Our findings of very good participant adherence and very few side effects supports the feasibility and tolerance of our ES protocol. A future large RCT should take into consideration the possibility that daily iES could irritate the vaginal lining.

The strengths of this study include; Randomization to intervention group; use of trained, blinded assessors; reproducible, individually-tailored intervention protocols; very good participant adherence; feedback that all subjects would recommend their therapy type to others; and use of reliable and valid manometry measurements. Whilst measurement of the strength of a PFM contraction using vaginal palpation [17] has demonstrated moderate to very good intra-examiner reliability, inter-examiner reliability has been found to be only fair [29]. Vaginal palpation of the PFMs is a commonly used method to assess PFM function in physiotherapy. However, manometry has been found to be more reliable, valid and sensitive to measure muscle strength, and there is often a poor agreement between testers when comparing vaginal palpation with manometry (45–47%) [16, 29]. Our study also clearly describes how facilitation techniques were undertaken with PFMT during treatment sessions.

A limitation of all pilot studies, including ours, is the small sample size. As anticipated, there were too few participants to be able to determine if iES is a more effective treatment than PFMT in women with weak PFM. Our primary aim was to determine how many participants we would require to detect a difference between groups in a full-scale RCT. Had the differences between groups been greater, we would not have required as many as 95 subjects in each group in a future study. If we had included a non-treatment control group, we may have found that the two intervention groups achieved a significant improvement compared to the control group. In a future large-scale RCT it may also be beneficial to record stage of POP using POP-Q.

## Conclusion

This study demonstrates that both iES and PFMT are feasible interventions in women with weak PFM. Women in both groups increased their PFM strength and reported improvements in symptoms of POP and urinary incontinence. As anticipated, it was not possible to determine if iES was a more effective therapy given the small sample size. To detect a difference between groups, we would have required 95 women in each group.
